# Pleomorphic viruses establish stable relationship with marine hyperthermophilic archaea

**DOI:** 10.1093/ismejo/wrae008

**Published:** 2024-01-23

**Authors:** Diana P Baquero, Eduardo A Bignon, Mart Krupovic

**Affiliations:** Institut Pasteur, Université Paris Cité, Archaeal Virology Unit, Paris 75015, France; Institut Pasteur, Université Paris Cité, Archaeal Virology Unit, Paris 75015, France; Institut Pasteur, Université Paris Cité, Archaeal Virology Unit, Paris 75015, France

**Keywords:** pleomorphic viruses, marine hyperthermophilic archaea, hydrothermal vents, Archaeoglobales, marine viruses, virus evolution, archaeal viruses

## Abstract

Non-lytic viruses with enveloped pleomorphic virions (family *Pleolipoviridae*) are ubiquitous in hypersaline environments across the globe and are associated with nearly all major lineages of halophilic archaea. However, their existence in other ecosystems remains largely unknown. Here, we show that evolutionarily-related viruses also infect hyperthermophilic archaea thriving in deep-sea hydrothermal vents. *Archaeoglobus veneficus* pleomorphic virus 1 (AvPV1), the first virus described for any member of the class *Archaeoglobi*, encodes a morphogenetic module typical of pleolipoviruses, including the characteristic VP4-like membrane fusion protein. We show that AvPV1 is a non-lytic virus chronically produced in liquid cultures without substantially affecting the growth dynamics of its host with a stable virus-to-host ratio of ~1. Mining of genomic and metagenomic databases revealed broad distribution of AvPV1-like viruses in geographically remote hydrothermal vents. Comparative genomics, coupled with phylogenetic analysis of VP4-like fusogens revealed deep divergence of pleomorphic viruses infecting halophilic, methanogenic, and hyperthermophilic archaea, signifying niche separation and coevolution of the corresponding virus-host pairs. Hence, we propose a new virus family, “*Thalassapleoviridae*,” for classification of the marine hyperthermophilic virus AvPV1 and its relatives. Collectively, our results provide insights into the diversity and evolution of pleomorphic viruses beyond hypersaline environments.

The *Pleolipoviridae* family includes genetically diverse archaeal viruses with enveloped pleomorphic virions, which encapsidate either single-stranded or double-stranded DNA genomes of 7–17 kb [^1–3^]. All known pleolipoviruses share a conserved block of genes encoding the morphogenetic module, including two major structural proteins and a putative nucleoside triphosphatase (NTPase) [2, 3]. The hallmark of pleolipoviruses is the spike protein VP4 (here we use HRPV1 protein nomenclature), which functions in host recognition and fusion of the viral and cellular membranes during virus entry [4, 5]. VP4 is thus far exclusive to pleolipoviruses and displays a unique structural fold, not observed in fusogens of other known viruses [4].

Pleolipoviruses have been isolated on haloarchaeal hosts from solar salterns and hypersaline lakes across the globe [^6–8^]. Furthermore, pleolipoviruses commonly encode integrases and are readily detected as proviruses integrated into the host chromosomes in a wide range of haloarchaeal species [^9–11^]. Accordingly, pleolipoviruses represent one of the most widely distributed and abundant groups of haloarchaeal viruses. Recently, proviruses encoding pleolipovirus VP4-like fusogens were detected in the genomes of methanogenic archaea of the orders *Methanonatronarchaeales* and *Methanomassiliicoccales* [12]. Here, we extend the host range of pleolipoviruses to anaerobic hyperthermophilic archaea of the class *Archaeoglobi*, common inhabitants of the deep-sea hydrothermal vents.

While studying extracellular cytochrome nanowires produced by the hyperthermophilic archaeon *Archaeoglobus veneficus* SNP6 [13], we noticed that the strain produces pleomorphic virus-like particles (VLPs) of ∼53 nm in diameter (*n* = 43; Fig. 1A; for details see Supplementary Methods). Analysis of the *A. veneficus* genome (NC_015320) revealed the presence of a putative provirus of 17.9 kbp flanked by direct repeats of 27 nucleotides, corresponding to putative attachment (*att*) sites (Fig. S1), which we refer to as *Archaeoglobus veneficus* pleomorphic virus 1 (AvPV1, see below). One of the *att* sites overlapped a *tRNA-Gly* gene, whereas the other one was adjacent to the integrase gene. The identification of a putative VP4-like membrane fusion protein, a signature protein of pleolipoviruses, in the provirus genome (Fig. 1B, Table S1), suggests that the observed VLPs could be produced by the identified provirus. We next confirmed that the virus is active and is released into the cell-free culture supernatants of *A. veneficus* by performing the polymerase chain reaction (PCR) analysis with primers targeting the excised and circularized form of the predicted provirus (Figs 1C, S1 and S2).

**Figure 1:**
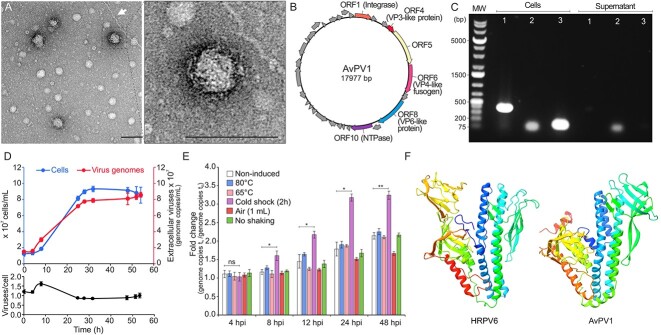
Characterization of the AvPV1; (A) electron micrographs of pleomorphic VLPs negatively stained with 2% uranyl acetate (scale bar, 100 nm); the arrow indicates an *A. veneficus* extracellular cytochrome nanowire [13]; (B) genome map of AvPV1. The ORFs are represented with arrows indicating the direction of transcription; (C) detection of AvPV1 in both cell and supernatant fractions in cultures of *A. veneficus* SPN6 by PCR; the experimental approach used is depicted in the schematic of Fig. S1; the agarose gel electrophoresis displays the PCR-amplified products: Lane 1, provirus integrated within the host chromosome (PCR amplification across the *attL* site, 412 bp); Lane 2, the excised and circularized form of the AvPV1 genome (PCR amplification across the reconstituted attV site, 108 bp); Lane 3, amplified fragment of the 16S rRNA gene of *A. veneficus* SNP6, 138 bp; (D) cell growth and virus production; the number of cells was estimated through a Thoma cell counting chamber over a period of 56 h; the titer of AvPV1 genomes in the supernatant of liquid cultures was assessed by qPCR using primers targeting the circular form of the virus genome; error bars represent standard deviation from three independent measurements; (E) virus genome fold change after induction using different stressor agents; the number of virus genomes in the supernatant was assessed by qPCR; samples were taken 0, 4, 8, 12, 24, and 48 h postinduction; error bars represent standard deviation from two independent measurements; stars indicate the significance levels based on the two-tailed *t*-test; ns, not significant; the *P* values are .5511, .0495, .0383, .0111, and .0083 from left to right; (F) comparison of the published crystal structure of the fusion protein of the haloarchaeal pleolipovirus HRPV6 (PDB: 6QGL) [4] to the AlphaFold2 structural model for the VP4-like fusion protein of AvPV1; terminal ends containing the transmembrane domains were trimmed for the convenience of presentation; protein structures are colored using the rainbow scheme from blue (N-terminus) to red (C-terminus).

We then evaluated the growth dynamics of *A. veneficus* and AvPV1 production over time. Similar to some other non-lytic archaeal viruses [8, 14, 15], AvPV1 was chronically produced at low rates without major adverse effects on the growth of its host (Fig. 1D). The virus production was most active during the exponential growth phase and ceased when the cells reached stationary phase. The virus-to-host ratio fluctuated around 1 (Fig. 1D), mirroring the recent metagenome-derived estimates across ecosystems [16]. To study whether this equilibrium can be destabilized, we challenged *A. veneficus* cultures with different environmental stressors. A modest but significant increase of ∼2-fold was only observed when cultures were exposed to a cold shock of 4°C for 2 h (Fig. 1E), a physiologically relevant factor in deep-sea ecosystems. These results suggest that the virus has evolved toward a stable relationship with its host, but this balance can be altered by changing environmental conditions.

The genome of AvPV1 contains 31 open reading frames (ORFs) (Fig. 1B, Table S1 in Data S1). BLASTP analysis showed that only two of the encoded proteins display significant similarity (E-value≤1e-5) to proteins of other known viruses. However, more sensitive profile–profile comparisons with HHpred revealed that AvPV1 carries a block of genes characteristic of pleolipoviruses. In particular, ORF4 and ORF6 encode homologs of the two major structural pleolipovirus proteins, VP3-like integral membrane protein and VP4-like fusogen, respectively (Table S1 in Data S1). Structural modeling of the AvPV1 VP4-like protein confirmed that it has the same V-shaped fold as the fusogens of bona fide pleolipoviruses (Fig. 1F, Fig. S3A). In addition, ORF8 and ORF10 encode homologs of the conserved HRPV1 protein ORF6 of unknown function and the putative NTPase, respectively (Fig. 1B, Table S1 in Data S1). Similar to gammapleolipovirus His2 [2], AvPV1 appears to encode a second divergent copy (ORF5) of a putative spike/fusion protein (HHpred probability of 87.9%). Unfortunately, we could not obtain a reliable AlphaFold structural model for this protein, likely due to lack of homologous in databases. Overall, these results indicate that AvPV1 is distantly related to pleolipoviruses of halophilic archaea and is the first representative of this virus lineage associated with marine archaea.

To assess the diversity and distribution of AvPV1-like pleomorphic viruses in extreme geothermal environments, we searched for AvPV1 VP4-like homologs in the Whole Genome Shotgun and non-redundant protein sequence databases at the National Center for Biotechnology Information (NCBI) and the Integrated Microbial Genomes/Virus database using TBLASTN or BLASTP (E-value≤1e-5). The searches yielded 19 contigs originating from geographically remote hydrothermal vents. Eight of the contigs corresponded to complete or nearly complete viral genomes (Fig. 2A and B, Table S2 in Data S1, Data S2). Three of the complete viral genomes were detected as proviruses integrated in the genomes of *Archaeoglobus profundus* DSM 5631, *Geoglobus acetivorans* SBH6, and *Geoglobus ahangari* 234, suggesting that hyperthermophilic pleomorphic viruses are primarily associated with members of the class *Archaeoglobi* (Table S2 in Data S1). The identified viruses exhibit a similar arrangement of the core structural genes to that of AvPV1, but all of them lack the second copy of the spike protein gene (Fig. 2A). Similar to AvPV1, the VP4-like fusogen of the identified viruses also exhibits a V-shaped fold (Fig. S3B–D). There are notable differences between viruses associated with *Archaeoglobus* and *Geoglobus* hosts. In particular, the two groups of viruses encode at least two non-orthologous groups of integrases of the tyrosine recombinase superfamily. Furthermore, viruses of *Geoglobus* encode a putative rolling-circle replication endonucleases (RCRE) of the HUH superfamily, likely responsible for the genome replication initiation (Fig. 2A, Table S3 in Data S1). Although alphapleolipoviruses also replicate their genomes using the rolling-circle mechanism [17, 18], the RCRE encoded by *Geoglobus* viruses was most closely related to homologs from pGT5/pTP1 family plasmids of *Pyrococcus* and *Thermococcus* species [19, 20], which also inhabit hydrothermal vents, suggesting niche-enabled horizontal gene exchange between viruses and plasmids in deep-sea hydrothermal vents. Consistent with the high sequence divergence between the structural proteins of *Pleolipoviridae* and AvPV1-like viruses (Table S3 in Data S1, Fig. S4), whole-proteome-based phylogenomic analysis placed AvPV1-like viruses outside of the family *Pleolipoviridae* (Fig. S5). Thus, we suggest that AvPV1 represents a new virus family, tentatively named “*Thalassapleoviridae*” after Thalassa, the primordial Greek goddess of the sea.

**Figure 2:**
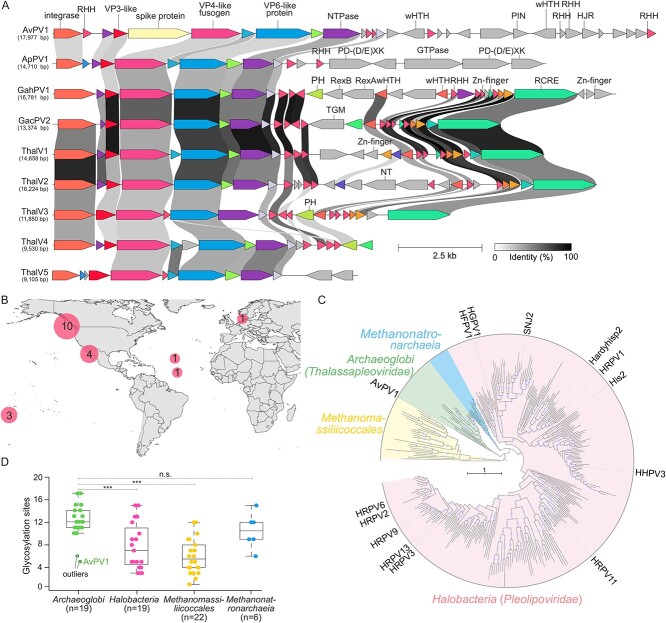
Characterization of hyperthermophilic pleomorphic viruses and their VP4-like membrane fusion proteins; (A) alignment of complete or nearly-complete genomes of pleomorphic viruses originating from marine deep-sea hydrothermal vents; ORFs are depicted by arrows that indicate the direction of transcription; functional annotations are depicted above the corresponding ORFs; homologous genes are shown using the same colors and are connected by shading in grayscale, with intensity reflecting the amino acid sequence identity; HJR, Holliday junction resolvase; NT, nucleotidyltransferase; PH, pleckstrin homology domain; PIN, PIN domain protein; RHH, ribbon-helix–helix protein; TGM, transglutaminase; wHTH, winged helix-turn-helix domain; Zn finger, zinc finger domain-containing protein; (B) geographical distribution of all hyperthermophilic pleomorphic viruses retrieved in this study; (C) maximum-likelihood analysis of VP4-like fusogens; clades of VP4 homologs encoded by viruses associated with different archaeal lineages are indicated with different colors; the scale bar represents the number of substitutions per site; circles at nodes indicate bootstrap support >90%; the complete version of the tree can be found in Fig. S6; (D) number of glycosylation sites (N-X-S/T, where X is any amino acid except proline) in VP4-like fusogens of pleomorphic viruses infecting *Archaeoglobi*, *Halobacteria*, *Methanonatronarchaeia*, and *Methanomassiliicoccales* hosts; stars indicate the significance levels based on the unpaired *t*-test; n.s., not significant; the *P* values are <.0001, <.0001, .2996 from left to right.

To study the relationships between pleomorphic viruses associated with hyperhalophilic (class *Halobacteria*), hyperthermophilic (*Archaeoglobi*), and methanogenic (*Methanomassiliicoccales* and *Methanonatronarchaeia*) archaea, we performed maximum likelihood phylogenetic analysis of the VP4-like fusogens and phylogenomic analysis based on the complete proteomes of the corresponding viruses. The four groups of viruses formed well-supported monophyletic clades in both analyses (Fig. 2C, Figs S5 and S6). In the absence of an objective outgroup, we rooted the tree with VP4 from *Methanomassiliicoccales*, a group of archaea assigned to a different phylum (*Thermoplasmatota*) than the other three archaeal lineages, all in *Halobacteriota*. *Archaeoglobi* VP4 homologs were at the base of the clade including *Halobacteria*, *Archaeoglobi*, and *Methanonatronarchaeia*, with viruses of haloarchaea and *Methanonatronarchaeia* forming monophyletic sister groups (Fig. 2C, Figs S5 and S6). The VP4 phylogeny is largely congruent with the archaeal species tree, suggesting divergence of the four groups of pleomorphic viruses concomitantly with the divergence of the corresponding host organisms, with no evidence of horizontal virus transfer and host-switching events between different archaeal lineages.

Comparison of structural models of the VP4-like fusogens from the four groups of viruses revealed a similar number of salt bridges and hydrophobic clusters (Fig. S7A and B, Table S4 in Data S1, Data S3). The homologs from viruses of hyperthermophilic and thermophilic *Archaeoglobi*, and *Methanonatronarchaeia*, respectively, had significantly higher numbers of predicted N-glycosylation sites (N-X-S/T, where X is any amino acid except proline) compared to viruses associated with *Halobacteria* and *Methanomassiliicoccales* which thrive at moderate temperatures (Figs 2D, S7C, Table S5 in Data S1). AvPV1 was an outlier and had fewer N-glycosylation sites (*n* = 5) compared to other *Archaeoglobi* viruses. We hypothesize that increased glycosylation is an adaptation to high-temperature environments ensuring VP4 stability. By contrast, compared to viruses of *Archaeoglobi*, VP4-like proteins of viruses associated with *Halobacteria* and *Methanonatronarchaeia*, both extreme halophiles, exhibited more extensive negative surface charge, a near-universal adaptation to hypersaline environments (Fig. S8). Thus, comparison of the VP4 structural models revealed differential adaption of the corresponding viruses to their respective environments.

AvPV1 is both the first relative of pleolipoviruses infecting hyperthermophilic archaea and also the first virus described for any member of the class *Archaeoglobi*. Our results demonstrate that pleomorphic viruses are globally distributed not only in hypersaline but also marine geothermal ecosystems, where they establish a stable relationship with their hosts.

## Supplementary Material

SI_ISMEJ_FINAL_wrae008

SI_Data_1_wrae008

SI_Data_2_wrae008

SI_Data_3_wrae008

## Data Availability

Genomes sequences of ApPV1, AvPV1, GacPV1, GahPV1, and ThalV2 were deposited to GenBank as Third Party Annotations (TPA) under the following accession numbers BK065154, BK065155, BK065156, BK065157, and BK065158, respectively. The genomes can be also found in the Supplementary data file 2.
