# Supplementary material for: Pleomorphic viruses establish stable relationship with marine hyperthermophilic archaea
Source: ISME J. 2024 Jan 23;18(1):wrae008. doi: 10.1093/ismejo/wrae008 (PMC10919331; doi:10.1093/ismejo/wrae008)
Supplement: SI_ISMEJ_FINAL_wrae008 [file si_ismej_final_wrae008.pdf]

Supplementary Information for

Pleomorphic viruses establish stable relationship with marine hyperthermophilic archaea

Diana P. Baquero[a], Eduardo A. Bignon[a], Mart Krupovic[a] *

[a] Institut Pasteur, Université de Paris, Archaeal Virology Unit, 75015 Paris, France

* Correspondence to: mart.krupovic@pasteur.fr

Keywords: pleomorphic viruses, marine hypethermophilic archaea, hydrothermal vents, *Archaeoglobales*, marine viruses, *Thalassapleoviridae,* virus evolution, archaeal viruses

Short title: Pleomorphic viruses of *Archaeoglobales*

List of contents:

1. *Supplementary Materials and Methods*
2. *Supplementary Figures*
3. *Supplementary Data files*
4. *Supplementary References*

1. MATERIAL AND METHODS

A. veneficus growth conditions

Archaeoglobus veneficus SNP6 cells [1] were purchased from the DSMZ culture collection. *A. veneficus* cultures (45-50 mL) were grown with agitation at 75°C in serum bottles under strict anoxic conditions in DSMZ medium 796, as described previously.

Propagation of virus particles

Cultures of *A. veneficus* (45-50 mL) were grown as described above and after two days of incubation, the cells were removed by low-speed centrifugation (Eppendorf F-35-6-30 rotor, 7,830 rpm, 20 min, 20°C). The cell-free supernatant was recovered, and virus-like particles were concentrated by ultracentrifugation (158,728 × *g*, 3 h, 15°C, Beckman 45 Ti rotor). After the run, the supernatant was carefully removed and the pellet was resuspended in 300 µL of phosphate-buffered saline (PBS) buffer.

Transmission electron microscopy (TEM)

5 µL of the samples were applied to carbon-coated copper grids and negatively stained with 2% uranyl acetate (wt/vol). Samples were imaged with the transmission electron microscope FEI Spirit Tecnai Biotwin operated at 120 kV. The dimensions of the negatively stained virus particles were determined using ImageJ [2].

Detection of AvPV1

PCR amplifications targeting the integrated and excised forms of AvPV1 were performed on washed *A. veneficus* cells and cell-free supernatants of *A. veneficus* cultures. PCR assays targeting the integrated form of the provirus (*attL* site) were performed using the following pair of primers: 1-F 5'-CTGACTACATTGCCTTCGCAC, 1-R 5'-CGAACAATAACGCCCGTGG. PCR amplifications detecting the excised and circularized form of the provirus were carried out using the following pair of primers: 2-F 5'-GTAATCTGGCAATCCTCCCGA, 2-R 5'-ATACGGCTTGACGCTGGAAA. PCR tests targeting a fragment of the 16S rRNA gene of *A. veneficus* SNP6 were used as control to confirm the absence of cells in the cell-free supernatant fraction (16s-F 5'-GTGCCTAAACAGCACCCCAT, 16s-R 5'-GATTTCCCGGGGGACTTACC). Cultures were grown as described above and the supernatant and cells were separated by low-speed centrifugation. Pelleted cells were resuspended in PBS buffer. The supernatant fraction was filtered through a 0.22 µm filter (Merck Millipore). PCR was performed using DreamTaq Green DNA Polymerase (Thermo Fisher Scientific) with the following steps: 95°C × 3 min followed by 35 cycles of 95°C ×30 s, 57°C ×30 s, and 75°C × 1 min, and a final extension step at 72°C × 10 min.

Virus quantification by qPCR

The number of viral genome copies was estimated by quantitative (q)PCR. Primers targeting the circularized form of AvPV1 (2-F 5'-GTAATCTGGCAATCCTCCCGA, 2-R 5'-ATACGGCTTGACGCTGGAAA) and 1 µL of the sample (cell-free supernatants) were mixed with the qPCR kit (Luna Universal qPCR Master Mix, New England Biolabs). qPCR was performed in a Bio-Rad CFX96 Touch Real-Time PCR Detection System with the following steps: 95°C × 1 min followed by 40 cycles of 95°C × 15 s, 57°C × 30 s, and 68°C × 20 s. A melting curve for the pair of primers and a calibration curve were performed.

Quantification of the virus production and cell growth

5 mL of exponentially growing cultures of *A. veneficus* were inoculated into 45 mL of the DSMZ medium 796 and incubated at 75°C with agitation (140 rpm). Aliquots were collected at defined time points to determine the amount of both cells and viral particles. The number of cells was estimated by direct counting through a Thoma chamber (depth 0.1 mm) whereas the virus was quantified by qPCR as described above. Experiments were conducted in triplicate.

Induction assays

To increase the number of viral particles produced by *A. veneficus* SNP6, we performed induction assays using various stressors such as variation of the growth temperature, cold shock, air exposure and, static conditions. All stressors were applied to cultures of *A. veneficus* grown for ~14 h. For temperature assays, cultures were placed into 60°C and 80°C shakers with continuous agitation throughout the experiment. In the case of cold shock, cultures were placed on ice for 2 hours. Subsequently, the cultures were returned to their optimal growth temperature with agitation. Given that *A. veneficus* is a strict anaerobe, 1 mL of air was injected into *A. veneficus* cultures. For the last condition, *A. veneficus* cultures were placed in an incubator at 75°C without agitation. Non-induced cultures were used as a control. Aliquots were collected under all tested conditions at different time points (0, 4, 8, 12, 24, 48 hours post-induction). The number of viral genome copies was estimated by qPCR, as described above. For all conditions and time points, the number of viral particles was normalized to the amount of viral genome copies at the time zero of the induction assay. All conditions were tested in duplicate.

Identification of the AvPV1 provirus in the genome of A. veneficus
Putative integration sites were established based on the presence of direct repeats in the host genome using Unipro UGENE [3]. Whenever a region flanked by direct repeats of minimum 20 bp and minimum distance between repeats of 5000 bp was identified, its genomic context was analyzed for the presence of tRNA genes. The *in silico*-translated proteins of the candidate regions were used as queries to search for distant homologs using HHpred [4] against PFAM (Database of Protein Families), PDB (Protein Data Bank), and CDD (Conserved Domains Database) databases. The results confirmed the presence of the AvPV1 provirus in the genome of *A. veneficus* (coordinates: 8107-26083) with genes characteristic of pleolipoviruses, including those encoding for VP4-like membrane fusion protein and VP3-like integral membrane protein.

Sequence database screening for AvPV1 VP4-like homologs
To identify viruses related to AvPV1, the AvPV1 VP4-like sequence was used as a query in BLASTP and TBLASTN searches (E-value≤1e-5) against the Whole Genome Shotgun (WGS) and non-redundant (nr) protein sequence databases at the NCBI, and the Integrated Microbial Genomes/Virus (IMG/VR) database [5]. Retrieved hits were manually analyzed, and sequences originating from hypersaline environments were excluded from subsequent analysis. The searches collectively yielded 19 contigs retrieved from globally distributed hydrothermal vents (Table S2).

Viral genome annotation and bioinformatics analysis
For all contigs/genomes retrieved, integration sites were defined based on the presence of direct repeats, which were searched for using Unipro UGENE [3]. ORFs were predicted using RAST v2.0 [6] and Prokka v.1.14.5 [7]. Each predicted ORF was manually validated for the presence of putative ribosome-binding sites upstream of the start codon. The *in silico*-translated protein sequences were used as queries to search for homologs in the non-redundant protein database at the NCBI with an upper threshold E- value of 1e-05. For the complete and nearly complete genomes, searches for distant homologs were performed using HHpred [4] against PFAM (Database of Protein Families), PDB (Protein Data Bank), and CDD (Conserved Domains Database) databases. Genomes of the identified viruses were compared using Clinker v.0.0.23 [8]. The sequences reported in this study have been deposited in GenBank. The accession numbers are pending attribution.

Structural models
Structural models of the VP4-like fusogens of AvPV1, ApPV1, GacPV1, and GahPV1 were downloaded from the AlphaFold database [9]. The AlphaFold2 structural models for the haloarchaeal pleolipovirus VP4 homologs were obtained from [10]. The models were visualized using ChimeraX [11]. Structural superposition of the AlphaFold2 models of VP4-like proteins of AvPV1, ApPV1, GacPV1, and GahPV1 with the published crystal structure of the HRPV6 fusion protein (PDB: 6QGL) [12] was performed using

the “matchmaker” algorithm of ChimeraX [11]. The numbers of salt bridges and hydrophobic clusters were determined for each structural model using the ProteinTools server [13].

Phylogenomic analyses

Phylogenomic analysis was conducted using the Genome-BLAST Distance Phylogeny (GBDP) method implemented in VICTOR, under settings recommended for prokaryotic viruses [14]. The proteomes of the newly described viruses were compared with those of haloarchaeal pleolipoviruses belonging to the three established genera of the family *Pleolipoviridae* and with pleomorphic viruses associated with *Methanomassiliicoccales* and *Methanonatronarchaeia* hosts [15]. The resulting intergenomic distances were used to infer a balanced minimum evolution tree with branch support via FASTME including SPR postprocessing for D6 formula. Branch support was inferred from 100 pseudo-bootstrap replicates each. For the maximum likelihood phylogenetic analysis, sequences of the VP4-like fusogens were retrieved from GenBank by performing 3 iterations of PSI-BLAST search queried with the HRPV6 fusogen sequence against the NCBI nr protein sequence database filtered to 70% identity. This dataset was supplemented with the homologs from hyperthermophilic viruses discovered in this study as well as those from recently described pleomorphic viruses associated with methanogenic archaea [15]. The sequences were aligned using MAFFT with -G-INS-1 option [16]. The positions with low information content were removed using trimal with the gap threshold of 0.2 [17]. The maximum likelihood phylogeny was inferred using IQ-TREE v1.6.12 [18]. The substitution model best fitting the data was identified with ModelFinder [19] and was Blosum62+F+R8. The branch support was assessed using SH-like approximate likelihood ration test (aLRT) with 1000 replicates. The tree was visualized with iTOL v6 [20].

Data availability

Genomes sequences of AvPV1, ApPV1, GahPV1, GacPV1, and ThalV2 were deposited to GenBank as Third Party Annotations (TPA) under the following accession numbers YYY, respectively. The genomes can be also found in the Supplementary data file 2.

2. SUPPLEMENTARY FIGURES

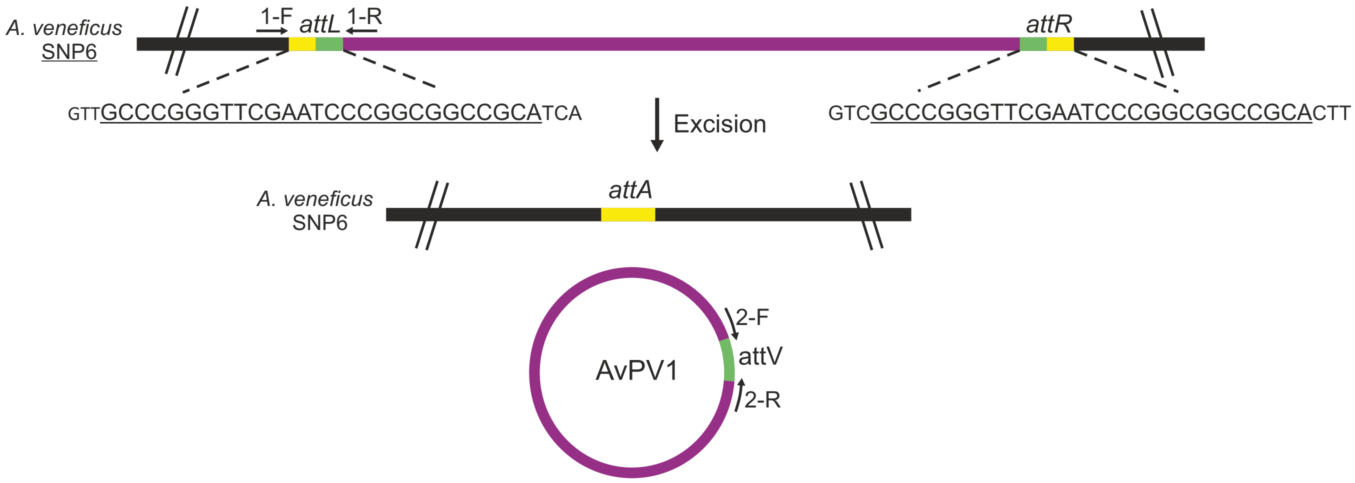

Figure S1. Schematic representation of the AvPV1 excision. The provirus is integrated in the host chromosome with the chimeric left (*attL*) and right (*attR*) attachment sites indicated in yellow and green. The excision of the provirus via site-specific homologous recombination between *attL* and *attR* produces a virus-free chromosome, reconstituting the original attachment site sequences (*attA*; yellow), and a circularized form of the virus genome carrying an identical attachment site (*attV*, green). Two sets of primers (indicated with small black arrows) were designed to detect the integrated and excised forms of the virus genome. Primers 1-F and 1-R target the virus genome integrated into the host chromosome *(attL)* (1-F 5'-CTGACTACATTGCCTTCGCAC, 1-R 5'-CGAACAATAACGCCCGTGG). The combination of primers 2-F and 2-R detects the excised and circularized form of the AvPV1 genome (2-F 5'-GTAATCTGGCAATCCTCCCGA, 2-R 5'-ATACGGCTTGACGCTGGAAA). The PCR results are shown in lanes 1 and 2 of **Fig. 1C**.

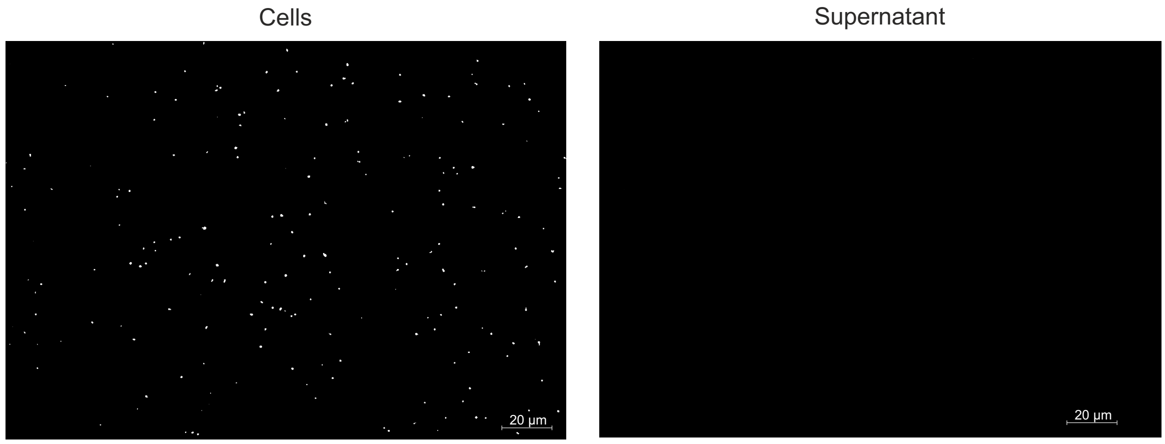

Figure S2. Fluorescence micrographs of both Nile-red-stained cellular and supernatant fractions. Bar, 20 µm.

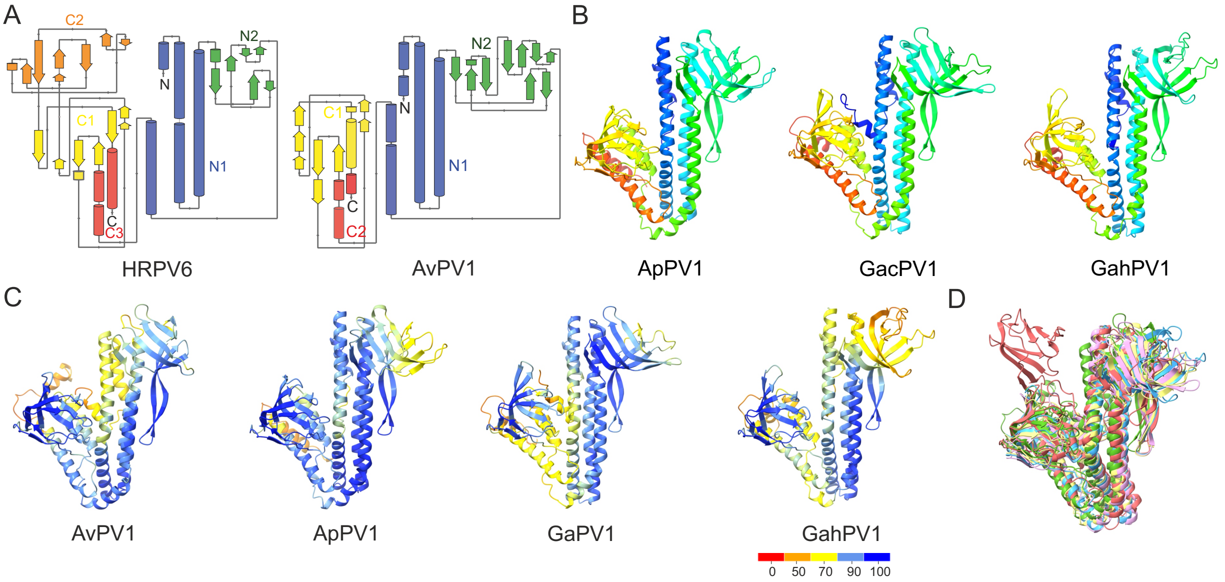

Figure S3. A, Topology representation of HRPV6 and AvPV1 VP4-like fusion protein colored by domains. HRPV6 representation is adapted from El Omari et al, 2019 [11]. **B,** AlphaFold2 structural models for VP4-like fusogens of ApPV1, GacPV1 and GahPV1 (all described in this study). Terminal ends were trimmed for the convenience of presentation. Protein structures are colored using the rainbow scheme from blue (N-terminus) to red (C-terminus). **C,** Quality assessment of the AlphaFold2 structural models of the VP4-like fusogens of AvPV1, ApPV1, GacPV1 and GahPV1. The residues are colored according to the pLDDT scores using ChimeraX [10]. Models with the pLDDT scores higher than 70 and considered to be of good quality. **D**, Structural superposition of the AlphaFold2 models of the VP4-like proteins from AvPV1 (green), ApPV1(yellow), GacPV1 (pink) and GahPV1 (light blue) with the published crystal structure of HRPV6 VP5 fusion protein (red) (PDB: 6QGL) [11]. Structures were superposed using the 'matchmaker' tool in ChimeraX [10].

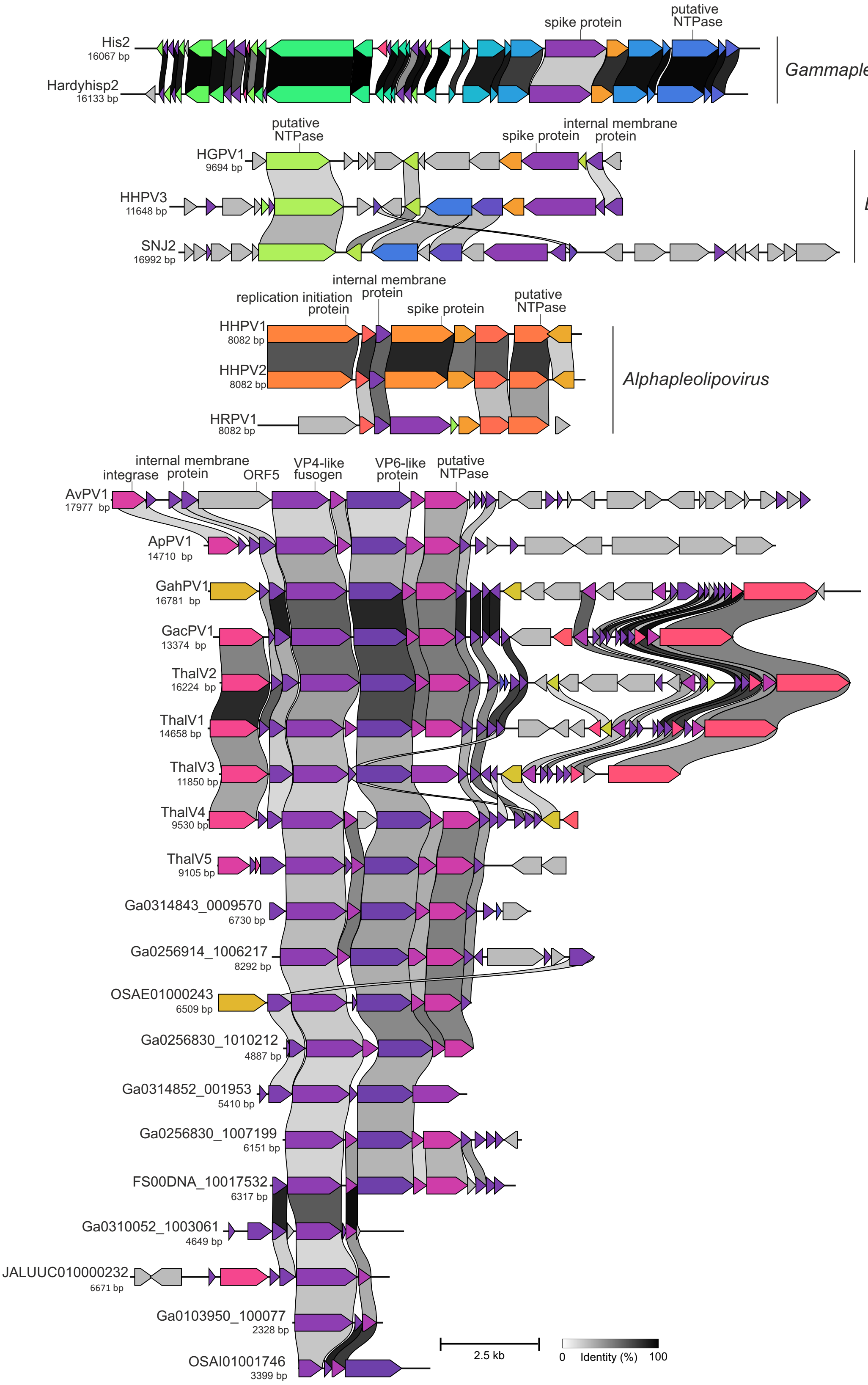

Figure S4. Genome comparison of all the sequences retrieved in this study containing AvPV1 VP4-like fusion protein. Representatives of the genera *Alphapleolipovirus*, *Betapleolipovirus* and *Gammapleolipovirus* were included in the analysis. The four set of viral sequences formed different modules. ORFs are represented by arrows that indicate the direction of transcription. Homologous genes are shown using the same colors and connected by shading in grayscale based on the amino acid sequence identity. Functional annotations of the predicted ORFs are depicted above the corresponding ORFs. The image was generated using Clinker v.0.0.23 [8]. The scale bar and precent identity shading are indicated in the bottom.

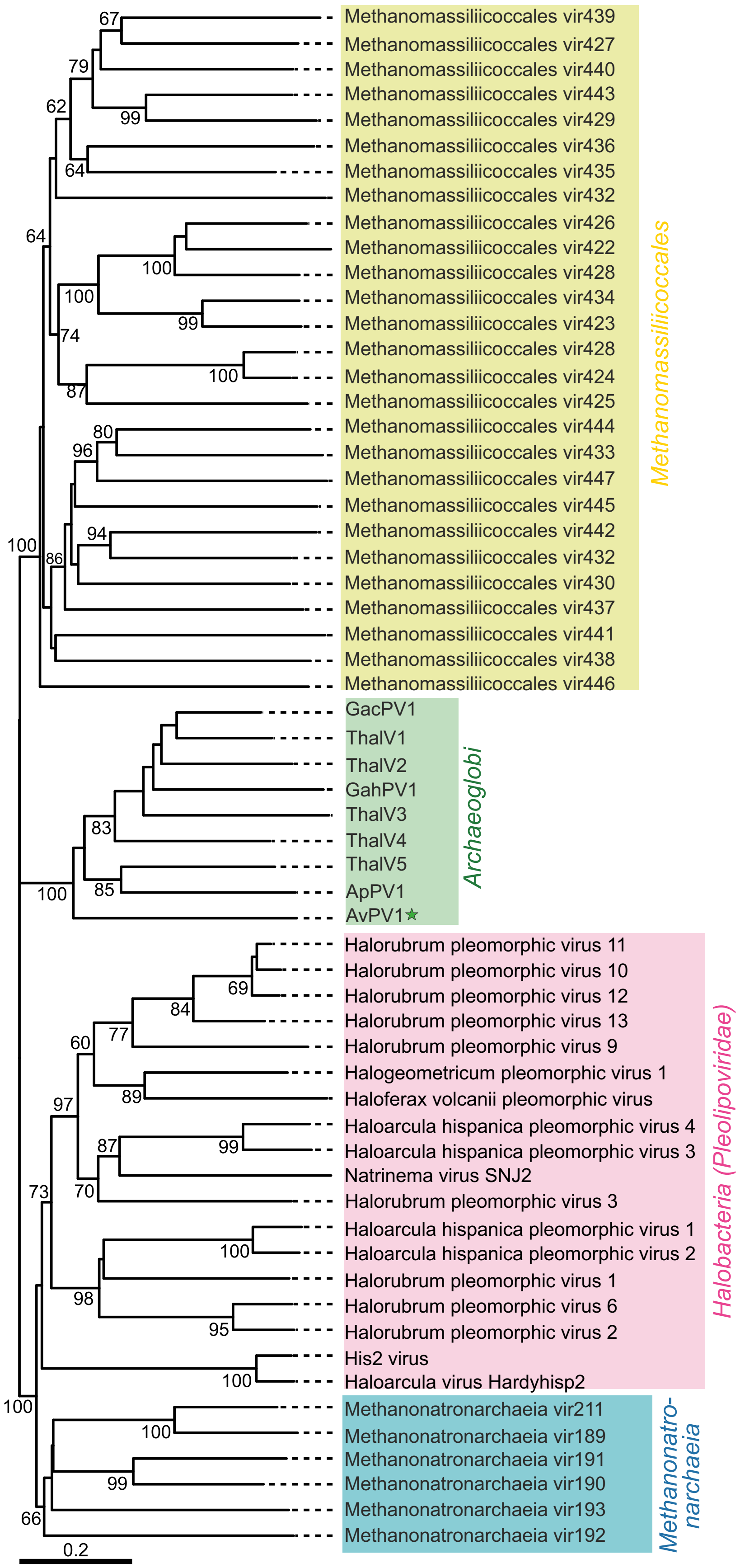

Figure S5. Inferred phylogenomic tree of pleomorphical viruses associated with hyperthermophilic (*Archaeoglobi*), halophilic (*Halobacteria*) and methanogenic (*Methanonatronarchaeia* and *Methanomassiliicoccales*) archaea based on whole genome VICTOR analysis at the amino acid level [12]. The tree is midpoint rooted and the branch length is scaled in terms of the Genome BLAST Distance Phylogeny (GBDP) distance formula D6. The position of AvPV1 is indicated with a green star.

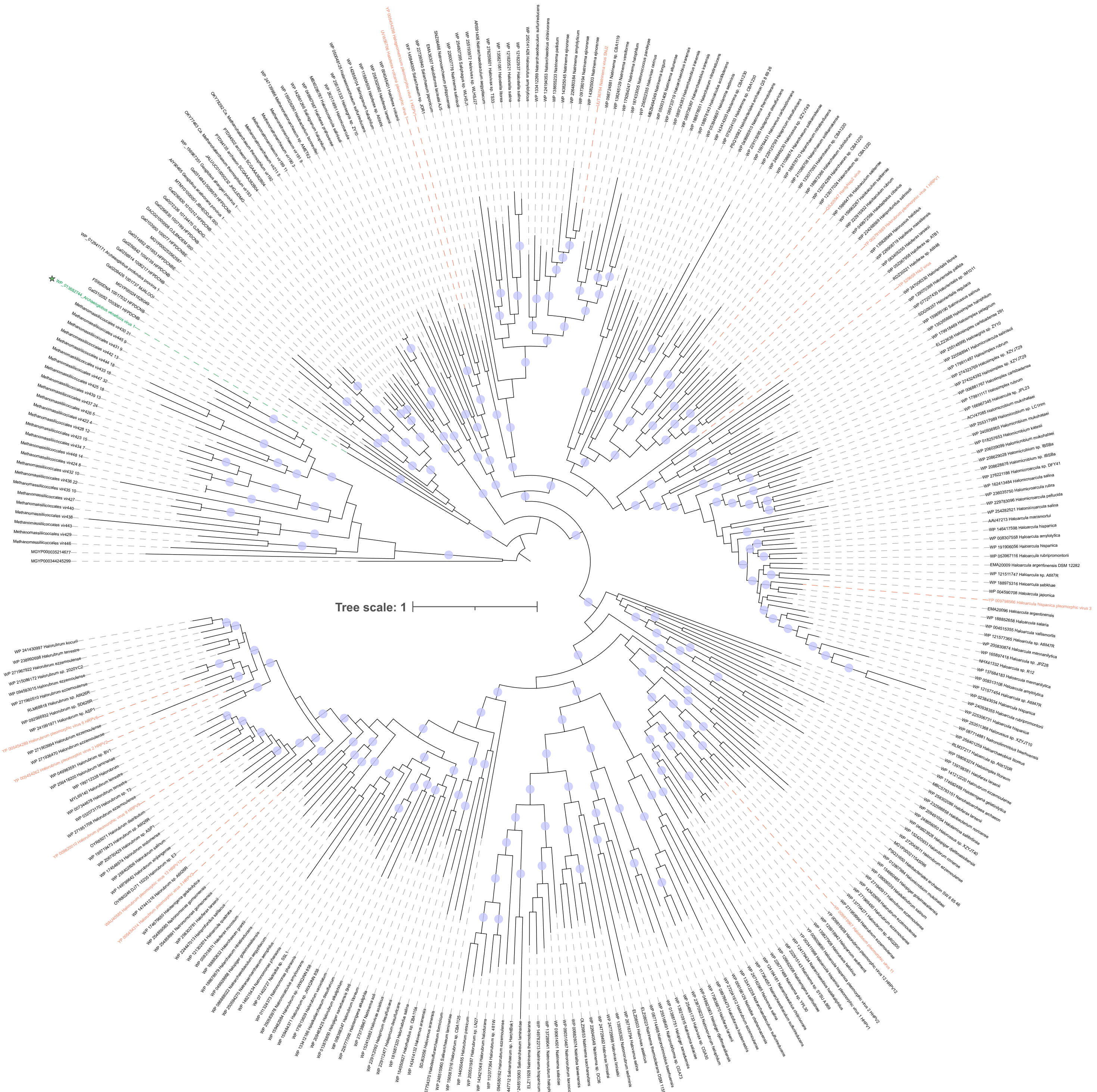

Figure S6. Complete maximum-likelihood analysis of VP4-like fusion membrane proteins. Clades of VP4 homologs encoded by viruses associated with different archaeal lineages are indicated with different colors. The position of AvPV1 is indicated with a green star. The scale bar represents the number of substitutions per site. Circles at nodes indicate bootstrap support >90%.

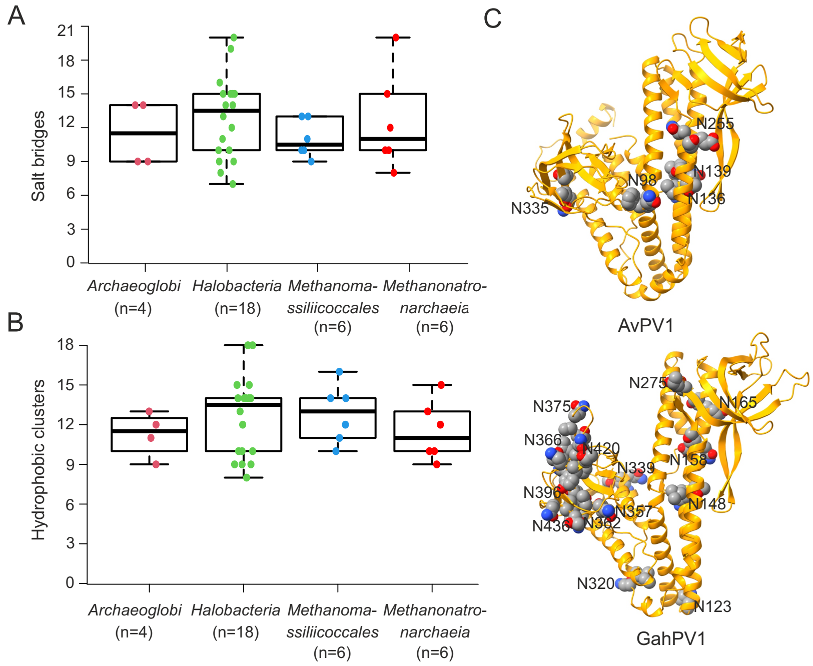

Figure S7. Comparison of VP4-like fusogens encoded by viruses infecting different archaeal hosts. **A**, Number of salt bridges for modeled VP4-like fusogens of pleomorphic viruses associated with hyperthermophilic (Archaeoglobi), halophilic (Halobacteria) and methanogenic (Methanonatronarchaeia and Methanomassiliicoccales) archaea. **B**, Number of hydrophobic clusters for modeled VP4-like fusogens of pleomorphic viruses infecting Archaeoglobi, Halobacteria, Methanonatronarchaeia and Methanomassiliicoccales. The numbers of salt bridges and hydrophobic clusters were determined for each structural model using the ProteinTools server [13]. **C**, Glycosylation patterns of the AvPV1 and GahPV1 VP4-like fusogens. The Asn residues in the predicted N-glycosylation sites are shown with a space-filling representation. The AlphaFold2 structural models for the haloarchaeal pleolipovirus VP4 homologs were obtained from [10].

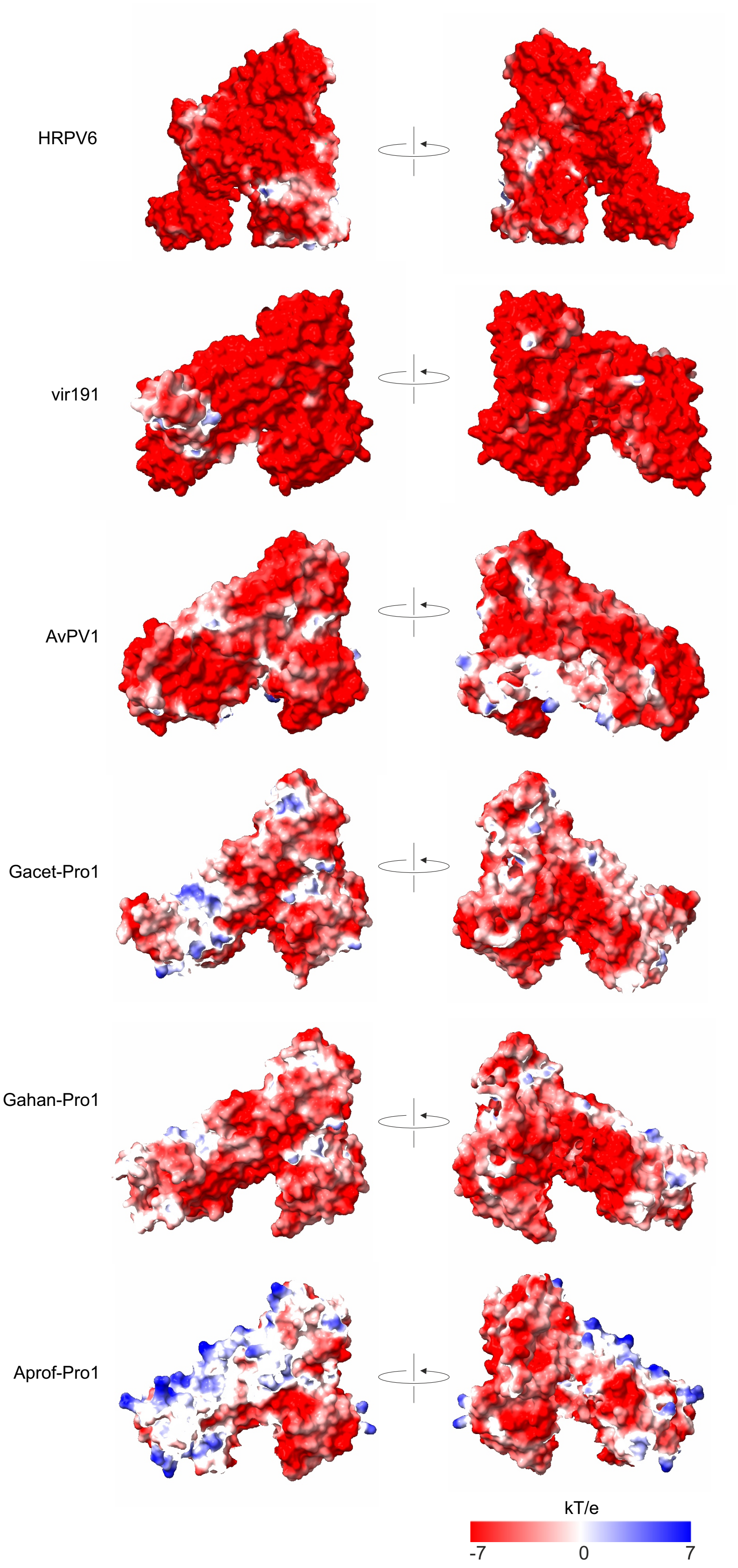

Figure S8. Electrostatic surface potentials of VP4-like proteins. Electrostatic surface potentials are colored red and blue for negative and positive charges, respectively, and white symbolizes neutral residues. The rendering was produced using ChimeraX. vir191 is associated with a *Methanonatronarchaeia* host.

3. SUPPLEMENTARY DATA FILES

Supplementary Data 1. Supplementary tables S1 to S5. All tables are included in a single XLSX spreadsheet.

Supplementary Data 2. "*Thalassapleoviridae*" genomes described and analyzed in this study. The archive contains annotated genomes in GenBank format.

Supplementary Data 3. AlphaFold2 structural models of all VP4-like proteins analyzed in Figure S7. Note that AlphaFold2 models for the haloarchaeal pleolipovirus VP4 homologs were obtained from [10]. The archive contains structural models in PDB format.

4. SUPPLEMENTARY REFERENCES

1. Huber H et al. *Archaeoglobus veneficus* sp. nov., a novel facultative chemolithoautotrophic hyperthermophilic sulfite reducer, isolated from abyssal black smokers. *Systematic and Applied Microbiology* 1997;**20**:374-80.
2. Schneider CA, Rasband WS, Eliceiri KW. NIH Image to ImageJ: 25 years of image analysis. *Nat Methods* 2012;**9**:671-5.
3. Okonechnikov K, Golosova O, Fursov M. Unipro UGENE: a unified bioinformatics toolkit. *Bioinformatics* 2012;**28**:1166-7.
4. Söding J, Biegert A, Lupas AN. The HHpred interactive server for protein homology detection and structure prediction. *Nucleic Acids Res* 2005;**33**:W244-8.
5. Camargo AP et al. IMG/VR v4: an expanded database of uncultivated virus genomes within a framework of extensive functional, taxonomic, and ecological metadata. *Nucleic Acids Res* 2023;**51**:D733-43.
6. Aziz RK et al. The RAST Server: rapid annotations using subsystems technology. *BMC Genomics* 2008;**9**:75.
7. Seemann T. Prokka: rapid prokaryotic genome annotation. *Bioinformatics* 2014;**30**:2068-9.
8. Gilchrist CLM. Chooi YH. clinker & clustermap.js: automatic generation of gene cluster comparison figures. *Bioinformatics* 2021;**37**:2473-5.
9. Varadi M et al. AlphaFold Protein Structure Database: massively expanding the structural coverage of protein-sequence space with high-accuracy models. *Nucleic Acids Res* 2022;**50**:D439-44.
10. Alarcón-Schumacher T, Lücking D, Erdmann S. Revisiting evolutionary trajectories and the organization of the Pleolipoviridae family. *PLoS Genet* 2023;**19**:e1010998.
11. Pettersen EF et al. UCSF ChimeraX: Structure visualization for researchers, educators, and developers. *Protein Sci* 2021;**30**:70-82.
12. El Omari K et al. The structure of a prokaryotic viral envelope protein expands the landscape of membrane fusion proteins. *Nat Commun* 2019;**10**:846.
13. Ferruz N, Schmidt S, Höcker B. ProteinTools: a toolkit to analyze protein structures. *Nucleic Acids Res* 2021;**49**:W559-66.
14. Meier-Kolthoff JP, Göker M. VICTOR: genome-based phylogeny and classification of prokaryotic viruses. *Bioinformatics* 2017;**33**:3396-404.
15. Medvedeva S et al. A compendium of viruses from methanogenic archaea reveals their diversity and adaptations to the gut environment. *Nat Microbiol* 2023;**8**:2170-82.
16. Katoh K et al. MAFFT version 5: improvement in accuracy of multiple sequence alignment. *Nucleic Acids Res* 2005;**33**:511-8.
17. Capella-Gutiérrez S, Silla-Martínez JM, Gabaldón T. trimAl: a tool for automated alignment trimming in large-scale phylogenetic analyses. *Bioinformatics* 2009;**25**:1972-3.
18. Trifinopoulos J et al. W-IQ-TREE: a fast online phylogenetic tool for maximum likelihood analysis. *Nucleic Acids Res* 2016;**44**:W232-5.
19. Kalyaanamoorthy S et al. ModelFinder: fast model selection for accurate phylogenetic estimates. *Nat Methods* 2017;**14**:587-9.
20. Letunic I, Bork P. Interactive Tree Of Life (iTOL) v5: an online tool for phylogenetic tree display and annotation. *Nucleic Acids Res* 2021;**49**:W293-6.
